# Patients with MDR-TB on domiciliary care in programmatic settings in Myanmar: Effect of a support package on preventing early deaths

**DOI:** 10.1371/journal.pone.0187223

**Published:** 2017-12-20

**Authors:** Pyae Phyo Wai, Hemant Deepak Shewade, Nang Thu Thu Kyaw, Khine Wut Yee Kyaw, Saw Thein, Aung Si Thu, Myo Minn Oo, Pyae Sone Htwe, Moe Myint Theingi Tun, Htet Myet Win Maung, Kyaw Thu Soe, Si Thu Aung

**Affiliations:** 1 International Union against Tuberculosis and Lung Disease (The Union), Mandalay, Myanmar; 2 International Union against Tuberculosis and Lung Disease (The Union), South-East Asia Office, New Delhi, India; 3 National Tuberculosis Programme, Ministry of Health and Sports, Myanmar; 4 Department of Medical Research (Pyin oo Lwin Branch), Ministry of Health and Sports, Myanmar; Instituto de Diagnostico y Referencia Epidemiologicos, MEXICO

## Abstract

**Background:**

The community-based MDR-TB care (CBMDR-TBC) project was implemented in 2015 by The Union in collaboration with national TB programme (NTP) in 33 townships of upper Myanmar to improve treatment outcomes among patients with MDR-TB registered under NTP. They received community-based support through the project staff, in addition to the routine domiciliary care provided by NTP staff. Each project township had a project nurse exclusively for MDR-TB and a community volunteer who provided evening directly observed therapy (in addition to morning directly observed therapy by NTP).

**Objectives:**

To determine the effect of CBMDR-TBC project on death and unfavourable outcomes during the intensive phase of MDR-TB treatment.

**Methods:**

In this cohort study involving record review, all patients diagnosed with MDR-TB between January 2015 and June 2016 in project townships and initiated on treatment till 31 Dec 2016 were included. CBMDR-TBC status was categorized as “receiving support” if project initiation in patient’s township was before treatment initiation, “receiving partial support” if project initiation was after treatment initiation, and “not receiving support” if project initiation was after intensive phase treatment outcome declaration. Time to event analysis (censored on 10 April 2017) and cox regression was done.

**Results:**

Of 261 patients initiated on treatment, death and unfavourable outcomes were accounted for 13% and 21% among “receiving support (n = 163)”, 3% and 24% among “receiving partial support (n = 75)” and 13% and 26% among “not receiving support (n = 23)” respectively. After adjusting for other potential confounders, the association between CBMDR-TBC and unfavourable outcomes was not statistically significant. However, when compared to “not receiving support”, those “receiving support” and “receiving partial support” had 20% [aHR (0.95 CI: 0.8 (0.2–3.1)] and 90% lower hazard [aHR (0.95 CI: 0.1 (0.02–0.9)] of death, respectively. This was intriguing. Implementation of CBMDR-TBC coincided with implementation of decentralized MDR-TB centers at district level. Hence, patients that would have generally not accessed MDR-TB treatment before decentralization also started receiving treatment and were also included under CBMDR-TBC “received support” group. These patients could possibly be expected to sicker at treatment initiation than patients in other CBMDR-TBC groups. This could be the possible reason for nullifying the effect of CBMDR-TBC in “receiving support” group and therefore similar survival was found when compared to “not receiving support”.

**Conclusion:**

CBMDR-TBC may prevent early deaths and has a scope for expansion to other townships of Myanmar and implications for NTPs globally. However, future studies should consider including data on extent of sickness at treatment initiation and patient level support received under CBMDR-TBC.

## Introduction

Multidrug-resistant/rifampicin-resistant tuberculosis (MDR-TB/RR-TB) is a public health burden worldwide with an estimated 580,000 cases and 250,000 deaths in 2015 [1]. Globally, the MDR-TB treatment success rate was 52% for the 2013 cohort. The loss to follow-up and death contribute to the majority of unsuccessful treatment outcome [2]. Death during treatment is seen in 13% of all MDR-TB patients registered for treatment [3].

Myanmar is one of the 30 high MDR-TB burden countries in the world. Based on the recent drug resistant survey (2012–13), five percent of new patients and 27% of previously treated patients have MDR-TB[4]. In 2015, National Tuberculosis Programmer (NTP) reported that of the estimated 9000 cases, 2,793 MDR-TB cases were diagnosed and 2,207 were enrolled for treatment in the same year. [5,6].

The treatment and care of MDR-TB is provided according to World Health Organization (WHO) recommended programmatic management of DR-TB (PMDT) model since 2011 [7]. Baseline investigations and treatment initiation are done at DR-TB treatment centers followed by domiciliary care in the community by a directly observed treatment (DOT) provider (DOT at the patient’s residence) that extends to 20 months. The MDR-TB treatment success rate (TSR) of those initiated on MDR-TB treatment in 2012 and 2013 was 79% and 83% respectively. This was higher than WHO 2015 TSR target of at least 75% [8].

By 2020, Myanmar targets to enroll all MDR-TB patients on treatment within two weeks of their diagnosis and provide comprehensive patient support package to enable treatment success rates of >80% [5] To achieve this, the International Union against Tuberculosis and Lung Disease (The Union) in collaboration with NTP started the community-based MDR-TB care (CBMDR-TBC) project in upper Myanmar since 2015 with funding from Global Fund (GF) and Three Millennium Development Goal Fund (3 MDGF).

Patients with MDR-TB diagnosed / registered under PMDT in project townships received support package under CBMDR-TBC through the project staff, in addition to the domiciliary care provided by PMDT staff. Trained community volunteers and project focal nurses (exclusively for MDR-TB) provided psychosocial and socio-economic support to patients and family members after MDR-TB diagnosis up to treatment initiation and completion under the guidance of NTP township TB team.

Phase-wise implementation of project in Upper Myanmar between 2015 and 2016 provided us a unique opportunity to assess the impact of this project. There is no published literature on effect of a support package (CBMDR-TBC in our case) to reduce deaths and unfavourable outcomes in the context of domiciliary care through PMDT. Therefore, as a first ever study, we aimed to assess whether the Union’s CBMDR-TBC project prevented deaths and unfavourable outcomes during intensive phase of MDR-TB treatment.

## Methods

### Study sesign

This is a retrospective cohort study involving record review.

### Setting

#### General setting

Myanmar is a lower middle income country [9] in south-east Asia region with a population of 51 million and predominantly mountainous in upper Myanmar, plain and delta region in middle and lower Myanmar [10]. It is administratively divided into states/regions (n = 15) followed by districts (n = 67) and townships (n = 330). Under the National Tuberculosis Program, there are TB centers at central level and systematically decentralized to state /region level, district level and township level[4].

#### PMDT in Myanmar

Patients with presumptive MDR-TB **(Table 1)** are referred from the township TB center to the nearest district TB center with Xpert MTB/Rif diagnostic facility. Each Xpert MTB/Rif facility has a laboratory register. A line list of presumptive MDR-TB register is maintained at the township level.

**Table 1:** Criteria to identify presumptive MDR-TB under programme setting who were referred to X-pert MTB/RIF testing facilities, Myanmar, 2015–16.

1	All previously treated TB patients
2	All new smear positive TB patients
3	All non-convertor TB patients (whose sputum result is still positive at the end of intensive phase)
4	HIV (+) TB patients
5	TB patients with past history of close contact with an MDR-TB patient and
6	TB patient with diabetes mellitus

DR-TB–multi drug-resistant tuberculosis, HIV–human immunodeficiency virus, TB—tuberculosis

All patients diagnosed as RR-TB by Xpert MTB/RIF are assumed as MDR-TB and started on second line treatment immediately. In selected cases (presumed to be having a low-risk of MDR-TB), an initial positive result is reconfirmed by a repeat Xpert MTB/Rif MTB/RIF. If needed, final confirmation is done with line probe assay (LPA) or culture and drug susceptibility test (DST) using Mycobacteria growth indicator tube (MGIT) liquid system (7).

After the patient is diagnosed with MDR-TB, the respective township TB team is informed. The township TB team includes township medical officer, township TB coordinator, basic health staff (BHS) and laboratory technician. The BHS (a nurse) provides care, including morning DOT, as per PMDT guidelines. The BHS is also responsible for implementation of other health programme related activities (in addition to coordinating TB related activities).

After the patient principally agrees to undertake MDR-TB treatment through DOT for at least 20 months, the patient is referred to the nearest MDR-TB treatment center (at district level) for baseline measurements (body weight, height, blood pressure) and baseline investigations followed by registration and treatment initiation. Baseline investigations include test for HIV, Hepatitis-B, Hepatitis-C, blood glucose, complete hemogram, liver function test, renal function test, ECG, thyroid function test, psychosocial assessment, hearing and ophthalmic assessment. The total duration of treatment is 18–22 months and consists of 6–8 months of intensive phase with five drugs (Amikacin, Pyrazinamide, Levofloxacin, Ethionamide, Cycloserine) followed by 12–14 months of continuation phase with four drugs (Pyrazinamide, Levofloxacin, Ethionamide, Cycloserine). Patient treatment card and MDR-TB treatment register are maintained at the MDR-TB treatment center and patient has a treatment booklet. All services including baseline and follow-up laboratory tests are provided free of cost [5]. By April 2016, all townships were covered under PMDT with a MDR-TB center in each township.

#### Community-based MDR-TB care (CBMDR-TBC) project

**Fig 1:**
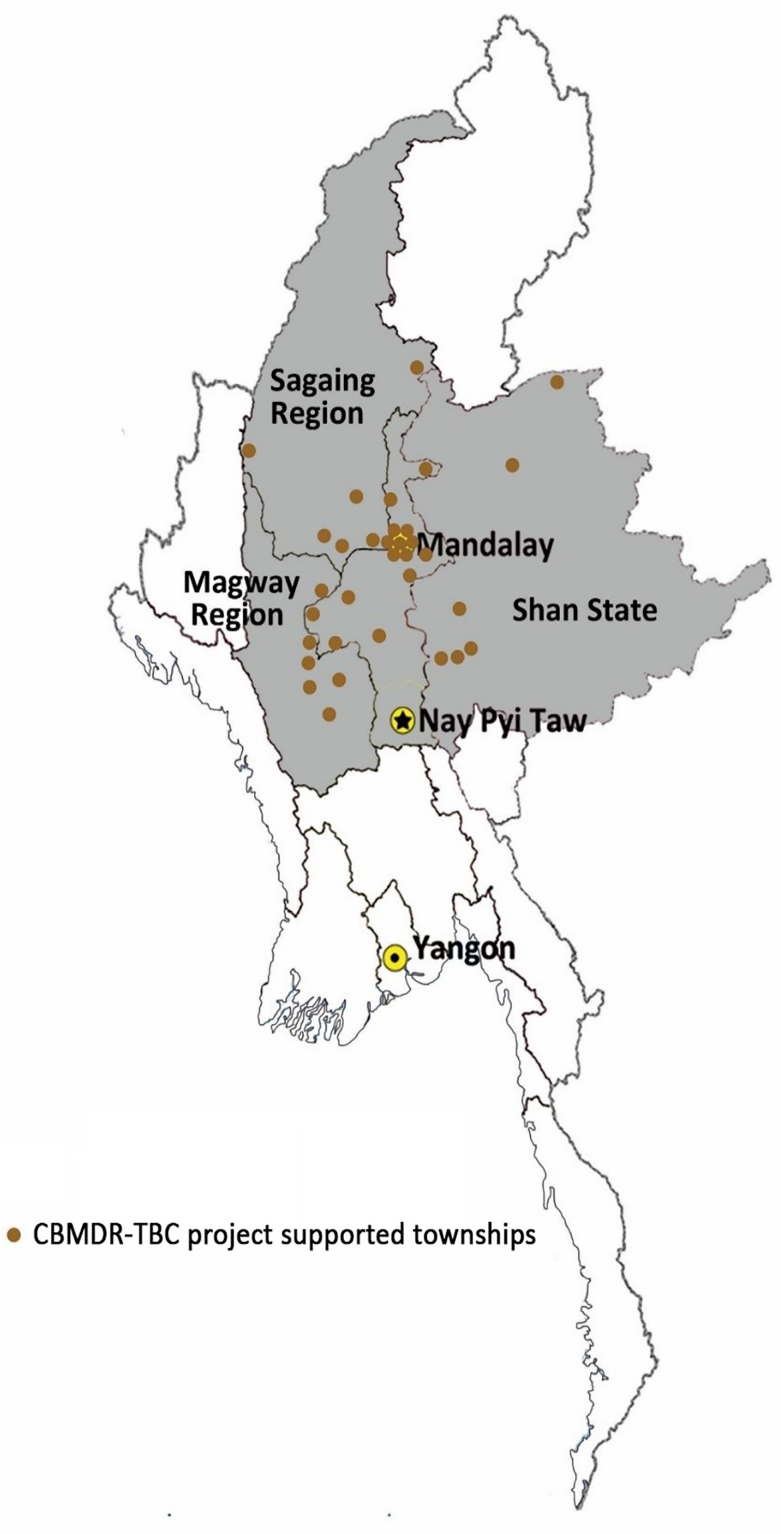
Map of Myanmar showing 33 community-based MDR-TB care project (CBMDR-TBC) supported townships across four states/regions of Upper Myanmar, 2015–16.

The Union’s CBMDR-TBC project supports PMDT in 33 townships (selected after consultation with NTP), across four states/regions in upper Myanmar since January 2015 **(Fig 1).** The project was implemented phase wise across the 33 townships between January 2015 and June 2016. Once the project implementation began in a particular township, all old and newly diagnosed patients received care under the project.

Each project township has a project focal nurse under supervision of the township TB team who exclusively works for MDR-TB. The project focal nurse ensures the implementation of the care package under PMDT as mentioned in **Table 2**. The nurse also identifies and trains a volunteer who lives close to the patient and acts as evening DOT provider. These volunteers help the patients get access to treatment and follow-up specimen transportation. The BHS continues to provide injection and morning DOT as per PMDT guidelines. The complementary support provided by the CBMDR-TBC project is summarized in **Table 3**. During 2015–16, of 49 Xpert MTB/Rif machines in the country, 12 were in 33 CBMDR-TBC townships.

**Table 2:** Package of support to all patients with MDR-TB under NTP’s PMDT in Myanmar, 2015–2016 [5].

	**Before treatment initiation**
1	Initial home visit and pretreatment counselling including the nature of medicines to be taken, the treatment process and the necessity of directly observed treatment (DOT) to monitor the treatment and offer regular support by the township medical officer, township TB coordinator, Basic Health Staff (BHS). This includes a written informed consent signed by the patient
2	Base-line investigations at the MDR-TB treatment center
3	Transport of sputum to the MDR-TB treatment center (for confirmation, if required)
	**After treatment initiation**
1	Daily morning injections and morning DOT by basic health staff
2	Treatment adherence counseling to patient and family member
3	Household and close contact investigation
4	Management of minor side effects of treatment at township TB center and timely referral to MDR-TB center (district level)
5	Monthly sputum smear examination at township TB center, sputum culture examination (on 3,5,8,12,14,16,18,20 months of treatment) and regular follow-up visits at DR-TB center
6	30 USD per month each to patient and DOT provider provided at township level
7	Nutritional support in the form of monthly ration of one nutrition powder package, rice (25 kg), beans 3.6 kg, oil 1.8 kg and salt 0.375 kg provided during visit to MDR-TB center for monthly follow-up

MDR-TB–multi drug resistant tuberculosis, NTP–national tuberculosis programme, PMDT–programmatic management of drug resistant tuberculosis, DOT–directly observed treatment

**Table 3:** Support package by the community-based MDR-TB care (CBMDR-TBC) project in Myanmar, 2015–16.

	Focal point nurse exclusively for MDR-TB care at each project township to support
1	Existing PMDT package as summarized in **Table 2**
2	Recruit and train a volunteer for evening DOT once patient starts treatment
3	Community mobilization by providing health education to the patient and their family members and neighbours
4	Pre-treatment support: 30 USD /month (for a maximum of 4 months) for patients with intent to reduce, to some extent, their expenses in lodging during visit to nearest DR-TB center, some ancillary drugs not provided by PMDT
5	Volunteer support to access care and follow up investigations
6	30 USD per month treatment provision allowance to the community volunteer under CBMDR-TBC
7	Psychosocial support through reassuring the patient to finish the whole course of treatment and counselling to patient, family members and neighbour

MDR-TB–multi drug resistant tuberculosis, PMDT–programmatic management of drug resistant tuberculosis, DOT–directly observed treatment, CBMDR-TBC–community-based MDR-TB care

Routine monitoring includes submission of monthly reports by volunteers to project focal nurse and then by the project focal nurses to project manager (one manager is assigned for every eleven townships) which are then forwarded to the monitoring and evaluation unit of The Union Office.

### Study participants

All patients diagnosed with MDR-TB between January 2015 and June 2016 in 33 CBMDR-TBC project townships of upper Myanmar were identified. Records of all Xpert MTB/Rif, LPA and MGIT tested positive patients were extracted from the 12 Xpert MTB/Rif facility laboratory registers and upper Myanmar TB center located in Mandalay. After removal of duplicates each study participant was given a unique identifier which was a combination of Xpert MTB/Rif facility code, Xpert MTB/Rif laboratory number and year. Date of diagnosis was defined as the date of Xpert MTB/Rif, LPA or MGIT test results. Earlier date was used in case of more than one test results.

Patients initiated on treatment until 31 December 2016 were included in the study. Entry of the patient into the cohort was based on the date of treatment initiation (01 January 2015 to 31 Dec 2016), while date of intensive phase treatment outcome or date of censoring (10 April 2017) whichever was earlier was the end date in the cohort.

### CBMDR-TBC exposure ascertainment and intensive phase treatment outcomes

To study the effect of CBMDR-TBC project on unfavourable intensive phase treatment outcomes, we categorized patients as: “not receiving support”, “partially receiving support” and “receiving support”. Date of field level initiation of the project in a township was taken as project initiation date. The patients were categorized as “not receiving support” if the date of project initiation in patient’s township was after the outcome date; as “partially receiving support” if the date of project initiation in patient township’s was after treatment initiation date but before outcome date; and as “receiving support” if the date of project initiation was before treatment initiation date.

Operational definitions of intensive phase treatment outcomes are summarized in **Table 4** and were in line with the prevalent WHO recommendations during the study period [11].

**Table 4:** Operational definition of MDR-TB treatment outcomes at end of intensive phase, Myanmar (2015–16) [11].

The standard duration of Intensive phase applied by the National program is 6 months. For treatment failed, lack of conversion by the end of the intensive phase implies that the patient does not convert within the maximum duration of intensive phase applied by the programme. If no maximum duration is defined, an 8-month cut-off is proposed. For regimens without a clear distinction between intensive and continuation phase, a cut-off of 8 months after the start of treatment is suggested to determine when the criteria for Cured, Treatment completed and Treatment failed start to apply.	
**Smear/Culture conversion**	A patient who has completed intensive phase of MDR-TB treatment, and two consecutive smear/culture is negative at the end of the intensive phase
**Smear/Culture non conversion**	A patient who has completed intensive phase of MDR-TB treatment, and sputum culture is not converted at the end of the intensive phase
**Loss to follow up**	A patient whose treatment was interrupted for 2 consecutive months or more
**Died**	A patient who dies for any reason during the course of treatment
**Not evaluated**	A patient whose outcomes are not available at the end of eight months (includes transfer out patients whose outcomes are not available)
**Still on treatment**	A patient who is alive and taking treatment as on 10 April 2017, but has not completed 8 months of treatment and does not fit into any of the other outcome definitions
**Favourable outcomes**	includes smear/culture conversion and still on treatment
**Unfavourable outcomes**	includes smear/culture non-conversion, loss to follow-up, death and not evaluated

MDR-TB–multi drug resistant tuberculosis, PMDT–programmatic management of drug resistant tuberculosis

### Data variables, sources of data and data collection

Variables collected from the MDR-TB treatment registers were: age, sex, resident township/region name, Xpert MTB/Rif facility name, Xpert MTB/Rif laboratory number, date of diagnosis, date of treatment initiation, site of TB, past history of TB, HIV status, resistance pattern, diabetes status, hepatitis B and hepatitis C status, weight in kg, hemoglobin (g/dl), intensive phase treatment outcome and date. Distance between patient Township and MDR-TB treatment center was calculated using google maps (www.googlemaps.com). Date of project initiation in patient’s township was collected from CBMDR-TBC project records.

### Analysis and statistics

Data collected in structured data collection forms were single entered into EpiData entry software (version 3.1, EpiData Association, Odense, Denmark) at each MDR-TB treatment centers by research assistants between March and April 2017. Descriptive analysis (frequency, proportion, means (SD), median (IQR)) and generation of derived variables was done using EpiData analysis software (version 2.2.2.183, EpiData Association, Odense, Denmark). STATA (version 12.1, copyright 1985–2011 StataCorp LP USA, serial number: 30120504773) was used for time to event analysis and to identify factors associated with death and unfavourable intensive phase treatment outcomes.

#### Derived variables

Based on the dates of treatment initiation, project initiation and intensive phase treatment outcome, CBMDR-TBC status was categorized as “not receiving support”, “partially receiving support” and “receiving support”. Sputum smear/culture conversion and ‘still on treatment’ were categorized as favourable and death, loss to follow-up, sputum smear/culture non-conversion and ‘not evaluated’ were categorized as unfavourable treatment outcome **(Table 4)**.Time to initiate treatment (in days) was calculated from date of diagnosis and date of treatment initiation. Hemoglobin measurements were categorized into ‘anaemia status’ using the classification for Asian populations as per WHO recommendations [12]. Weight was categorized using the cut off 45 kilogram.

Under CBMDR-TBC status, “receiving support” and “receiving partial support” was the exposure of interest. Death and unfavourable outcomes during intensive phase was the outcome of interest which was summarized as proportion and incidence rate (number of events per 1000 person-days of follow-up)

Unadjusted analysis was done to determine the association (Hazard Ratio, HR) between the exposure of interest, other potential confounders and outcome of interest. Unadjusted Kaplan Meier curves were used to describe the outcome free survival over time: overall and stratified by CBMDR-TBC status. Age, sex, CBMDR-TBC status and variables with *p*-value of <0.2 in the unadjusted analysis were included (after ruling out multi-collinearity) in the Cox regression model (enter method). We assessed for proportional hazard assumption of the model by plotting the estimated survival curves using Cox model and Kaplan-Meier estimates **(S1 and S2 Figs)**. Unadjusted and adjusted HRs were reported with 95% confidence intervals (CI).

### Ethics

Ethics approval was received from Ethics Review Committee, Department of Medical Research, Ministry of Health and Sports, Myanmar (ERC No. 014216, dated 30^th^ January 2017) and the Ethics Advisory Group of International Union against Tuberculosis and Lung Disease (The Union), Paris, France (EAG No. 81/16, dated 1^st^ November 2016). Permission to conduct the study was granted from National Tuberculosis Programme, Ministry of Health and Sports, Myanmar. As the study involved analysis of secondary data from programme records, waiver for informed consent was sought and approved by the ethics committees.

## Results

### Baseline characteristics

A total of 261 patients were initiated on treatment. Baseline demographic characteristics have been summarized in **Table 5.** Of the total, 118 (45%) were of the age group 15–34 years; 176 (67%) were males and 159 (61%) from Mandalay region. Sixty four (25%) patients had a MDR-TB center in their township. Baseline clinical characteristics have been summarized in **Table 6.** Of the total, 30 (12%) were HIV positive, 256 (98%) had pulmonary TB and 115 (44%) had a weight of less than 45 kilogram. Days to treatment initiation was more than 14 days in 220 (86%) cases.

**Table 5:** Demographic characteristics of patients with MDRTB registered for treatment between January 2015 and June 2016 in 33 community-based MDR-TB care (CBMDR-TBC) project supported townships in Myanmar.

Characteristics		N	(%)
**Total**		**261**	**(100)**
Age (year)	< 15	1	(0.4)
	15–34	118	(45)
	35–54	103	(40)
	≥55	39	(15)
Sex	Male	176	(67)
	Female	85	(33)
Patient residence state/region	Mandalay	159	(61)
	Magway	26	(10)
	Sagaing	33	(13)
	Northern Shan	26	(10)
	Southern Shan	17	(7)
Distance from treatment facilities	Same township	64	(25)
	<100 km	125	(48)
	≥ 100 km	72	(27)

MDR-TB-multidrug resistance tuberculosis, CBMDR-TBC- community based multidrug resistance tuberculosis care

**Table 6:** Clinical characteristics of patients with MDRTB registered for treatment between January 2015 and June 2016 in 33 community-based MDR-TB care (CBMDR-TBC) project supported townships in Myanmar.

Characteristics		N	(%)
**Total**		**261**	**(100)**
Previously treated TB	Yes	232	(89)
	No	29	(11)
HIV status	Non-reactive	156	(60)
	Reactive	30	(12)
	Unknown	75	(29)
Registration group	Relapse	79	(30)
	Treatment after failure	121	(46)
	Treatment after default	11	(4)
	Treatment after second line	1	(0.4)
	New	28	(11)
	Other	20	(8)
	Not recorded	1	(0.4)
Resistance pattern	Resistance to first line	259	(99)
	Resistance to second line	1	(0.5)
	Not recorded	1	(0.5)
Site of TB	PTB	256	(98)
	EPTB	1	(0.5)
	Both	3	(1)
	Not recorded	1	(0.5)
Diabetes mellitus	No	35	(13)
	Yes	134	(51)
	Not recorded	92	(35)
Hepatitis B infection status	Positive	13	(5)
	Negative	246	(94)
	Not recorded	2	(1)
Hepatitis C infection status	Positive	10	(4)
	Negative	249	(95)
	Not recorded	2	(1)
Anaemia	No anaemia	87	(33)
	Anaemia	162	(62)
	Severe Anaemia	12	(5)
Weight in kg	<45	115	(44)
	≥45	129	(49)
	Unknown	17	(7)
Time in days from diagnosis to	<14	36	(14)
treatment initiation	14–49	125	(48)
	50–99	53	(20)
	≥100	47	(18)

MDR-TB-Multidrug resistance tuberculosis, TB–Tuberculosis; HIV–Human immunodeficiency virus

Based on CBMDR-TBC status, 163 (62%) received support under CBMDR-TBC and 75 (29%) received partial support. We also looked at the duration in days for which the patient received CBMDR-TBC: 178 (68%), 30 (12%) and 53 (20%) received care for more than four months, two to four months and less than two months respectively. **(Table 7)**

**Table 7:** Care under community-based MDR-TB (CBMDR-TBC) project and end intensive phase treatment outcomes among patient with MDR-TB registered for treatment between January 2015 and June 2016 in 33 CBMDR-TBC project supported townships in Myanmar.

Variable			N	(%)
Total			261	(100)
**CbMDR-TBC status**				
	**Receiving support**	Under care before treatment initiation	163	(62)
	**Receiving partial support**	Under care after treatment initiation	75	(29)
	**Not receiving support**	Not under care till declaration of outcomes	23	(9)
**Duration under CBMDR-TBC**				
		Under care ≥4months	178	(68)
		Under care 2–4 months	30	(12)
		Under care <2 months	53	(20)
**Treatment outcome**				
	**Favorable**		**200**	**(77)**
		Sputum/smear conversion	196	(75)
		End IP outcome not declared, not completed 8 months and still on treatment	4	(2)
	**Unfavorable**		**61**	**(23)**
		Sputum/culture non-conversion	4	(2)
		Death	26	(10)
		Loss to follow-up	4	(2)
		Not evaluated	27	(10)

MDR-TB—Multi drug resistant tuberculosis

### Intensive phase treatment outcomes

Sixty one patients (23%) had unfavourable treatment outcome: death and not evaluated contributing to 26 (10%) and 27 (10%) outcomes respectively. **(Table 7)**

### CBMDR-TB and intensive phase treatment outcomes

There were 47335 person-days of follow: it was 3702, 16037 and 27596 among “not receiving support”, “receiving partial support” and “receiving support” group.

**Fig 2:**
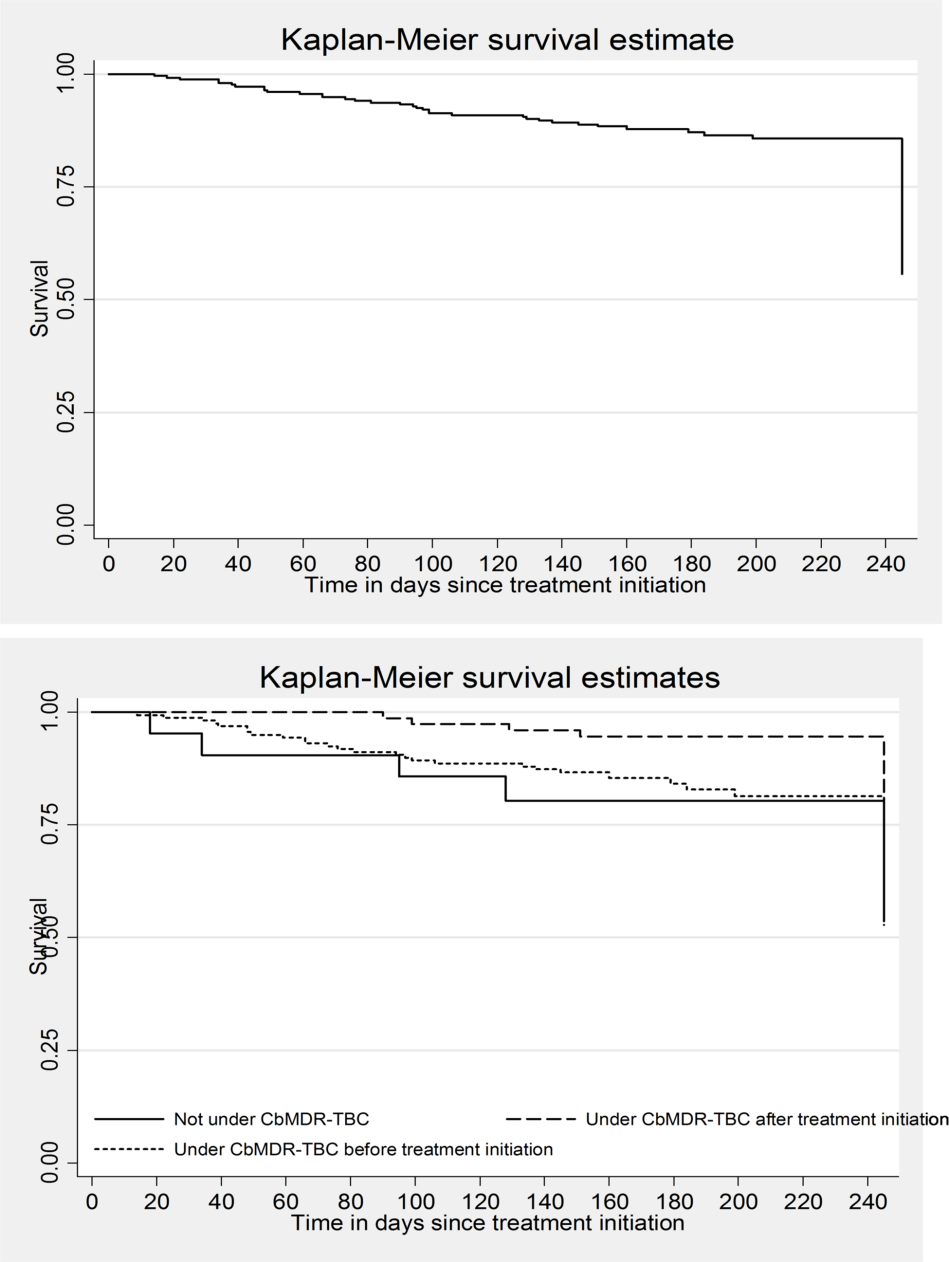
Unfavourable outcomes (smear/culture non-conversion, loss to follow-up, death and not evaluated) in intensive phase among patients with MDR-TB registered for treatment between January 2015 and June 2016 in 33 community-based MDR-TB care (CBMDR-TBC) project supported townships in Myanmar (all patients and by CBMDR-TBC status). *under CBMDR-TBC after treatment initiation (“receiving partial support”); under CBMDR-TBC before treatment initiation (“receiving support”); Log rank test p value = 0.14 (unadjusted).

The number (% (0.95 CI)) of unfavourable outcome among those “not receiving support (n = 23)”, “receiving partial support (n = 75)” and “receiving support (n = 163)” was 6 (26.1 (11.1, 48.7)), 16 (21.3 (13.0, 32.6)) and 38 (23.3 (17.2, 30.7)) respectively. Incidence rate (0.95 CI) for unfavourable outcomes was 1.3 (1.0, 1.6) per 1000 person-days of follow-up. Based on CBMDR-TBC status, incidence rate (0.95 CI) among those “not receiving support”, “receiving partial support” and “receiving support” was 1.6 (0.7, 3.6), 1.0 (0.6, 1.6) and 1.4 (1.0, 1.9) respectively per 1000 person-days of follow-up. The survival curves for unfavourable outcome; overall and stratified by CBMDR-TBC status, are depicted in **Fig 2**. The outcome free survival was better for those “receiving partial support” when compared to those “not receiving support”.

**Fig 3:**
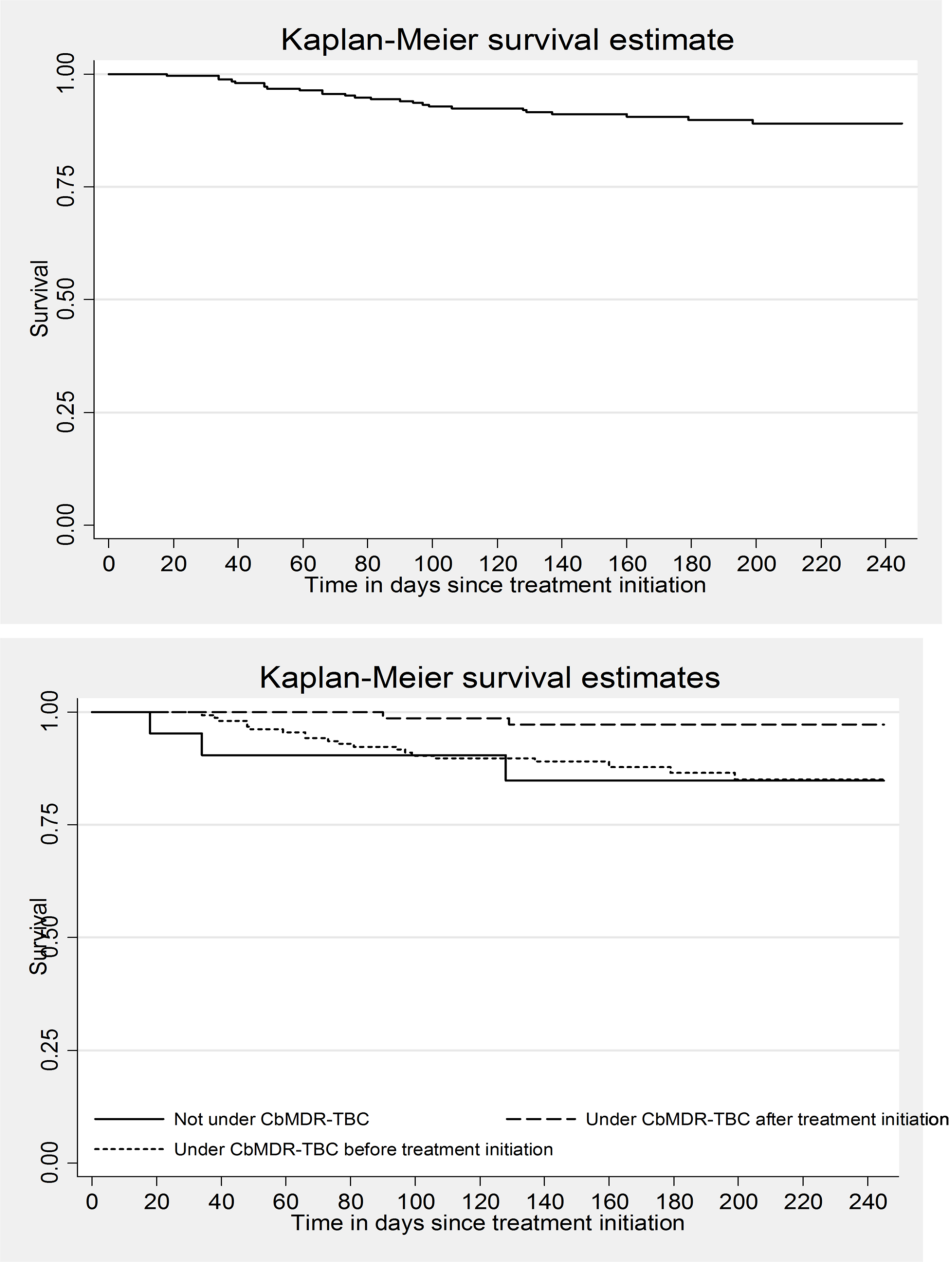
Death in intensive phase among patients with MDRTB registered for treatment between January 2015 and June 2016 in 33 community-based MDR-TB care (CBMDR-TBC) project supported townships in Myanmar (all patients and by CBMDR-TBC status). *under CBMDR_TBC after treatment initiation (“receiving partial support”); under CBMDR-TBC before treatment initiation (“receiving support”); Log rank test p value = 0.03 (unadjusted).

The number (% (0.95 CI)) of death among those “not receiving support (n = 23)”, “receiving partial support (n = 75)” and “receiving support (n = 163)” was 3 (12.7 (3.4, 34.7)), 2 (3 (0.5, 10.2)) and 20 (12.3 (7.8, 18.5)) respectively. Incidence rate (0.95 CI) for death was 0.5 (0.4, 0.8) per 1000 person-days of follow-up. Based on CBMDR-TBC status, incidence rate among those “not receiving support”, “receiving partial support” and “receiving support” was 0.8 (0.3, 2.5), 0.1 (0.0, 0.5) and 0.7 (0.5, 1.1) respectively per 1000 person-days of follow-up. The survival curves for death; overall and stratified by CBMDR-TBC status, are depicted in **Fig 3**. The death free survival was better for those “receiving partial support” when compared to those “not receiving support”.

For the independent predictive effect of CBMDR-TBC project on intensive phase treatment outcomes, age, sex, HIV status, anaemia and CBMDR-TBC status were added in a cox regression model. For the model predicting death, region was also included. Distance and duration of treatment were not included due to very high p value on unadjusted analysis. Diabetes was excluded because of high proportion of missing data. Unadjusted and adjusted HRs for unfavourable outcomes and death have been presented in **Tables 8 and 9** respectively.

**Table 8:** Risk factors for unfavorable treatment outcomes among patients with MDR-TB registered for treatment between January 2015 and June 2016 in 33 community-based MDR-TB care (CBMDR-TBC) project supported townships in Myanmar.

Characteristics		Total	Unfavourable Outcome		HR (0.95 CI)	aHR* (0.95 CI)
		N	n	(%)		
**Total**		**261**	61	(23)		
Age (year)	< 15	1	0	(0)	-	-
	15–34	118	20	(17)	**Ref**	Ref
	35–54	103	26	(25)	1.6(0.9–3.0)	1.7(0.9–3.2)
	≥55	39	15	(39)	2.0(1.1–4.1)	**2.2(1.0–4.5)^**
Sex	Male	176	36	(20)	**Ref**	**Ref**
	Female	85	25	(29)	1.2(0.7–2.1)	1.0(0.3–2.1)
HIV status	Reactive	30	12	(40)	1.9(0.9–3.9)	1.7(0.8,3.7)
	Unknown	75	21	(28)	1.6(0.9–2.9)	0.6(0.2,2.3)
	Non-reactive	156	28	(18)	**Ref**	**Ref**
Anaemia	No anaemia	87	16	(18)	**Ref**	**Ref**
	Anaemia	162	39	(24)	1.7(0.9–3.2)	**1.9(1.0–3.6)^**
	Severe Anaemia	12	6	(50)	3.9(1.5–10.1)	**2.7(1.0–7.1)^**
Weight in kg	<45	115	33	(29)	1.8(1.1–3.1)	**1.9(1.0–3.4)^**
	≥45	129	22	(17)	**Ref**	**Ref**
	Unknown	17	6	(35)	2.4(1.0–6.0)	**2.7(1.0–7.1)^**
CbMDR-TBC	Receiving support	163	39	(24)	0.9(0.4–2.3)	0.5(0.2–1.3)
	Receiving partial support	75	16	(21)	0.6(0.2–1.5)	0.9(0.3–2.1)
	Not receiving support	23	6	(26)	**Ref**	**Ref**

aHR: adjusted hazard ratio, CI: confidence interval

*aHR calculated using Cox regression (enter method): age, sex, CbMDR-TBC and variables with unadjusted p<0.2 were included in the regression model and shown in this table

^p<0.05

Model AIC / BIC 591.3 / 598.4

**Table 9:** Risk factors for death among patients with MDRTB registered for treatment initiation between January 2015 and June 2016 in 33 community-based MDR-TB care (CBMDR-TBC) project supported townships in Myanmar.

Characteristics		Total	Death		HR (0.95 CI)	aHR* (0.95 CI)
		N	n	(%)		
**Total**		**261**	26	(10)		
Age (year)	< 15	14	0	(0)	-	-
	15–34	118	5	(4)	**Ref**	**Ref**
	35–54	103	12	(12)	2.6(0.9–7.6)	2.4(0.8–7.1)
	≥55	39	9	(23)	5.9(2.0–17.7)	**8.3(2.6–26.8)^**
Sex	Male	176	16	(9)	**Ref**	Ref
	Female	85	10	(12)	1.2(0.5–2.6)	0.6(0.2–1.7)
Patient	Mandalay	159	18	(11)	**Ref**	**Ref**
Residence	Magway	26	4	(15)	1.4(0.4–4.2)	1.1(0.3–3.6)
	Sagaing	33	2	(6)	0.5(0.1–2.2)	0.5(0.1–2.2)
	Northern Shan	26	1	(4)	0.3(0.4–2.4)	0.2(0.0–1.9)
	Southern Shan	17	1	(6)	0.5(0.7–4.0)	0.9(0.1–7.3)
HIV status	Reactive	30	7	(23)	3.4(1.2–9.4)	2.6(0.8–8.6)
	Unknown	75	9	(12)	1.9(0.8–4.7)	2.2(0.8–5.8)
	Non-reactive	156	10	(6)	**Ref**	**Ref**
Anaemia	No anaemia	87	3	(3)	**Ref**	**Ref**
	Anaemia	162	19	(12)	5.4(1.3–23.2)	**5.7(1.2–26.7)^**
	Severe Anaemia	12	4	(33)	17.3(3.2–94.8)	**8.3(1.2–56.8)^**
Weight in kg	<45	115	17	(15)	2.7(1.1–6.5)	1.8(0.7–5.1)
	≥45	129	7	(5)	Ref	**Ref**
	Unknown	17	2	(12)	2.3(0.5–11.0)	3.8(0.7–20.3)
CbMDR-TBC	Receiving support	163	21	(13)	1.0(0.3–1.1)	0.8(0.2–3.1)
	Receiving partial support	75	2	(3)	0.2(0.0–1.1)	**0.1(0.0–0.9)^**
	Not receiving support	23	3	(13)	**Ref**	**Ref**

aHR: adjusted hazard ratio, CI: confidence interval

*adjusted hazard ratio (aHR) calculated using Cox regression (enter method): age, sex, CbMDR-TBC and variables with unadjusted p<0.2 were included in the regression model and shown in this table AIC / BIC: 265.7 / 272.8

^ p <0.05

After adjustment of potential confounders, when compared to “not receiving support”, those “receiving support” and “receiving partial support” had 50% [aHR (0.95 CI: 0.5(0.2–1.3))] and 10% lower hazard [aHR (0.95 CI: 0.9 (0.3–2.1)] of unfavourable outcome, respectively. However, this was not statistically significant. For death, “receiving support” and “receiving partial support” had 20% [aHR (0.95 CI: 0.8 (0.2–3.1)] and 90% lower hazard [aHR (0.95 CI: 0.1 (0.02–0.9)], respectively. The latter was statistically significant. **(Tables 8 and 9)**

Other independent predictors (risk factors) for unfavourable outcomes as well as death were age more than 55 years and anaemia. Weight less than 45 kilogram was also associated with unfavourable outcomes. **(Tables 8 and 9)**

## Discussion

### Summary of key findings

In the context of high MDR-TB treatment success through domiciliary care under PMDT in Myanmar, patients who received support through CBMDR-TBC midway during their treatment had lower deaths in intensive phase of treatment. However, if support through CBMDR-TBC was received before treatment initiation, the deaths were comparable with patients who did not receive any support at all. CBMDR-TBC did not have an effect on unfavourable outcomes during intensive phase. Treatment delay and distance between patient’s township and DR-TB treatment center were not associated with unfavourable outcomes.

### Strengths and limitations

This is first study from Myanmar and possibly worldwide, to determine the effect of a support package, complementing existing domiciliary care for MDR-TB under programme settings (PMDT), on early deaths and unfavourable outcomes in intensive phase. The study involved use of routine programmatic data; our findings reflect the ground reality. We followed the strengthening the reporting of observational studies in epidemiology (STROBE) guidelines to report our findings [13].

However, the study had some limitations as well. First, information on diabetes was missing for 35% of patients and height was not recorded. Therefore, we could not include diabetes status and body mass index variable in adjusted analysis. In addition to this, information on other patient level data (extent of exposure to CBMDR-TBC in the form of number of nurse /volunteer visits and extent of sickness at treatment initiation) and programmatic / health system level factors was not available as this was not collected routinely within the programme. This study was based on existing programmatic records; there might be some measurement and recording errors that are inherent to operational research and we do not have control over the numbers of participants in each group for comparison (there were 23 patients under ‘not receiving care’ group). Second, the date of project initiation was at the township level. We do not think that this will induce any clustering as all patients (including those already on treatment) were provided services once the project was implemented in a township. Third, if the township was large, there might be large margin of error depending where someone lived in that township. This error was possible as we did not consider the distance from actual patient residence. However this error was not expected to vary differentially among the CBMDR-TBC groups (‘receiving support’, ‘receiving partial support’ and ‘not receiving support’).

### Interpretation of findings

There were many programmatic relevant findings in our study.

First, receiving support beginning from a period before treatment initiation did not prevent deaths while receiving support beginning from anytime between treatment initiation and outcome declaration prevented deaths. This was intriguing as we expected even more deaths from being prevented in those receiving support beginning from a period before treatment initiation.

High attrition before treatment initiation among MDR-TB has been documented in Myanmar [4,5]. Implementation of CBMDR-TBC coincided with implementation of decentralized MDR-TB centers at district level. Hence, patients that would have generally not accessed MDR-TB treatment before decentralization of MDR-TB centers started receiving treatment and were also included under CBMDR-TBC “received support” group. These patients could possibly be expected to be sicker at treatment initiation than patients in other CBMDR-TBC groups. This could be the possible reason for nullifying the effect of CBMDR-TBC in “receiving support” group and therefore similar survival was found when compared to “not receiving support”.

Overall, the effect of CBMDR-TBC on early deaths could be due to more effective implementation of domiciliary care under PMDT through a focal nurse at township level who exclusively worked for MDR-TB. We are assuming that a patient after enrolment into CBMDR-TBC must have received all the benefits at all the time. Therefore, the estimates provided in our study are conservative and the true effect could be higher.

Second, the project did not have an effect on unfavourable treatment outcomes as a whole. One plausible reason for this may be the large proportion of “not evaluated” patients at the end of 8 months within the cohort (10%). These were the patients who completed 8 months of treatment without any unfavourable outcome, but the culture results were not available in time for declaring them as ‘culture converted’. Of these 27, eleven received partial CBMDR-TBC support and 14 received support throughout. Hence, the absence of effect of CBMDR-TBC on unfavourable outcomes in intensive phase could be a false negative result due to missing culture results in records.

Third, treatment delay was high as only 14% of the patients were initiated on treatment within 14 days of diagnosis. This delay was also not associated with early death or unfavourable intensive phase treatment outcomes. In 2016, a systematic review identified no published evidence linking delay in treatment initiation and MDR-TB outcomes[14]. Recently, a study from India has reported delayed treatment initiation (>30 days) as a risk factor for unfavourable outcomes [15].

Fourth, among all patients, twelve percent died or were lost to follow-up at the end of eight months. Low loss to follow up may be attributable to nutritional support provided to the patient. Monetary support under PMDT in the form of 30 USD per month provided each to BHS and patient for DOT provision and support respectively may also have a role to play. In India, among a cohort of patient enrolled in 2011–12 for MDR-TB care, nineteen percent of patients documented unfavourable end treatment outcomes occurred within six months of treatment [16].

Fifth, high unfavourable outcomes including death among those aged more than 55 years may be due to existing co-morbidities in this age group. Diabetes is one of them: we could not include it in the risk factor analysis due to large number of missing values. Like in our study, other risk factors like anaemia and low weight or body mass index have also been linked to unfavourable outcomes. [17]. A systematic review identified HIV as a factor for early deaths: it was not the case in our study[18].

### Policy implications

First, we recommend qualitative systematic enquiry to study patient and health-system related enablers and barriers for successful / unsuccessful treatment while receiving CBMDR-TBC support. This may also provide more insights as to why patients receiving CBMDR-TBC support before treatment initiation did not have the same effect on deaths as those receiving CBMDR-TBC midway during the treatment did.

Second, we recommend the follow-up of the same cohort till the end of treatment outcomes to determine the effect of CBMDR-TBC on end treatment outcomes. We may not get this opportunity in cohorts after this as all 33 townships in the CBMDR-TBC project have been covered under the project.

Last, the NTP may consider expansion of CBMDR-TBC to all townships. This has implications for preventing early deaths across Myanmar. Currently, The Union is one of the non-Government organizations supporting the PMDT in providing this support package. This also may have implications for other countries, especially those with poor MDR-TB outcomes. The same may be modified to suit each country’s setting and implemented with appropriate modifications.

## Conclusion

The Union’s community-based MDR-TB care project supported the existing domiciliary care provided under NTP in Myanmar. Under the project, a focal nurse in a township and a community volunteer (providing evening DOT) ensured timely initiation and adherence to MDR-TB treatment. Support through CBMDR-TBC after treatment initiation prevented early deaths during MDR-TB treatment. This may have scope for expansion to other the township of Myanmar and has implication for NTPs globally. In addition, age more than 55 years, anaemia and weight less than 45 kg were identified as predictors for unfavourable intensive phase outcomes including deaths. Further follow-up of the same cohort till end of MDR-TB treatment should be done to determine the effect of CBMDR-TBC on end treatment outcomes. However, future studies should consider including data on extent of sickness at treatment initiation and patient level support received under CBMDR-TBC.

## Supporting information

S1 Fig Assessment of proportional hazards assumption for CBMDR-TBC status for occurrence of ‘unfavourable intensive phase treatment outcome’ by plotting the estimated survival curves obtained using Cox model and Kaplan-Meier estimates* (Model presented in Table 8). *exp2 variable categorized as (Yes) under care before treatment initiation; (Partial care) under care after treatment initiation; and (No) Not under care till declaration of outcomes; Model AIC / BIC 591.3 / 598.4
(TIF)

S2 Fig Assessment of proportional hazards assumption for CBMDR-TBC status for occurrence of ‘death during intensive phase’ by plotting the estimated survival curves obtained using Cox model and Kaplan-Meier estimates (Model presented in Table 9). *exp2 variable categorized as (Yes) under care before treatment initiation; (Partial care) under care after treatment initiation; and (No) Not under care till declaration of outcomes; AIC / BIC: 265.7 / 272.8
(TIF)
