# Supplementary figures and images for: Patients with MDR-TB on domiciliary care in programmatic settings in Myanmar: Effect of a support package on preventing early deaths
Source: PLoS One. 2017 Dec 20;12(12):e0187223. doi: 10.1371/journal.pone.0187223 (PMC5737886; doi:10.1371/journal.pone.0187223)

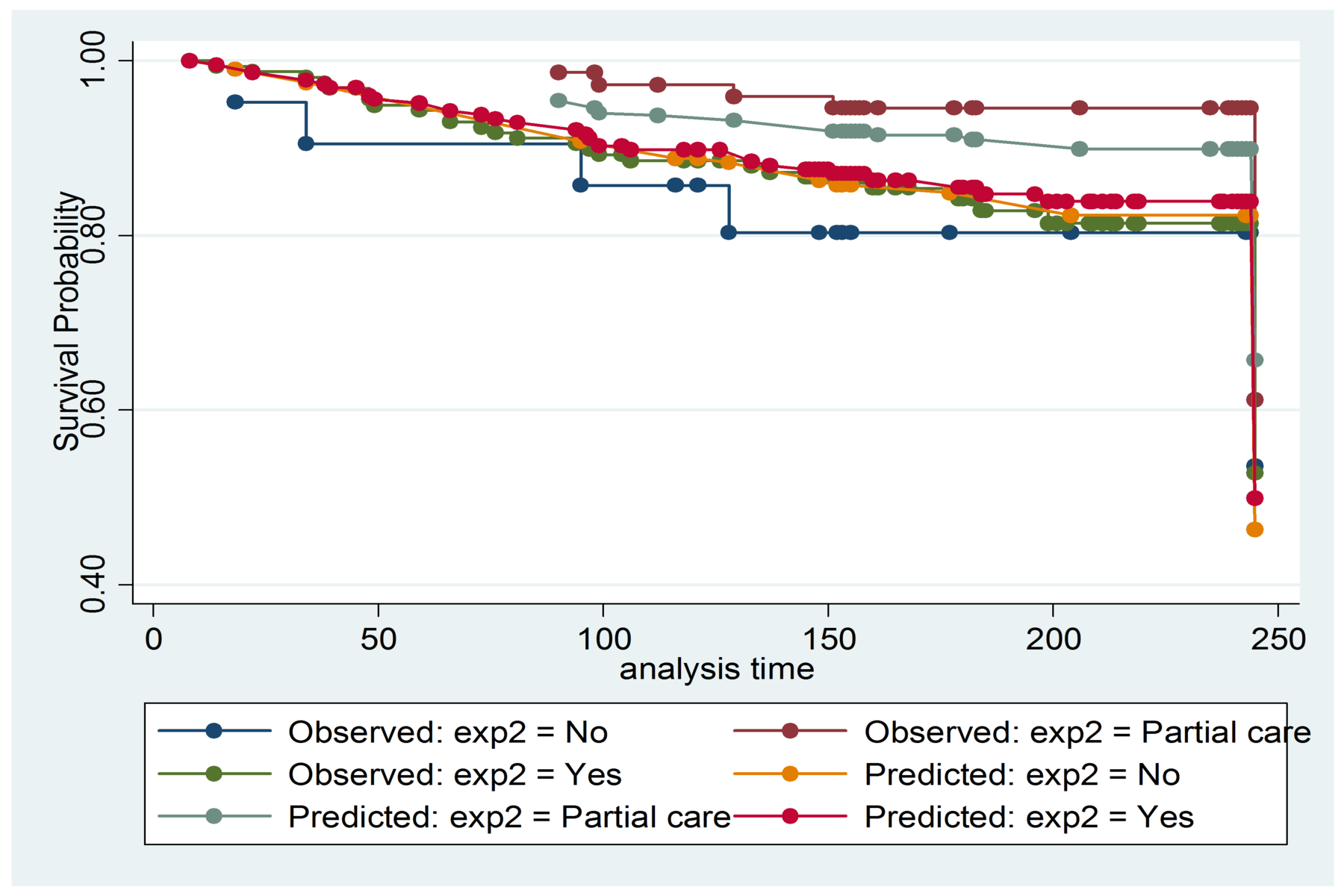

Supplement: S1 Fig — *exp2 variable categorized as (Yes) under care before treatment initiation; (Partial care) under care after treatment initiation; and (No) Not under care till declaration of outcomes; Model AIC / BIC 591.3 / 598.4 (TIF) [file pone.0187223.s001.tif]

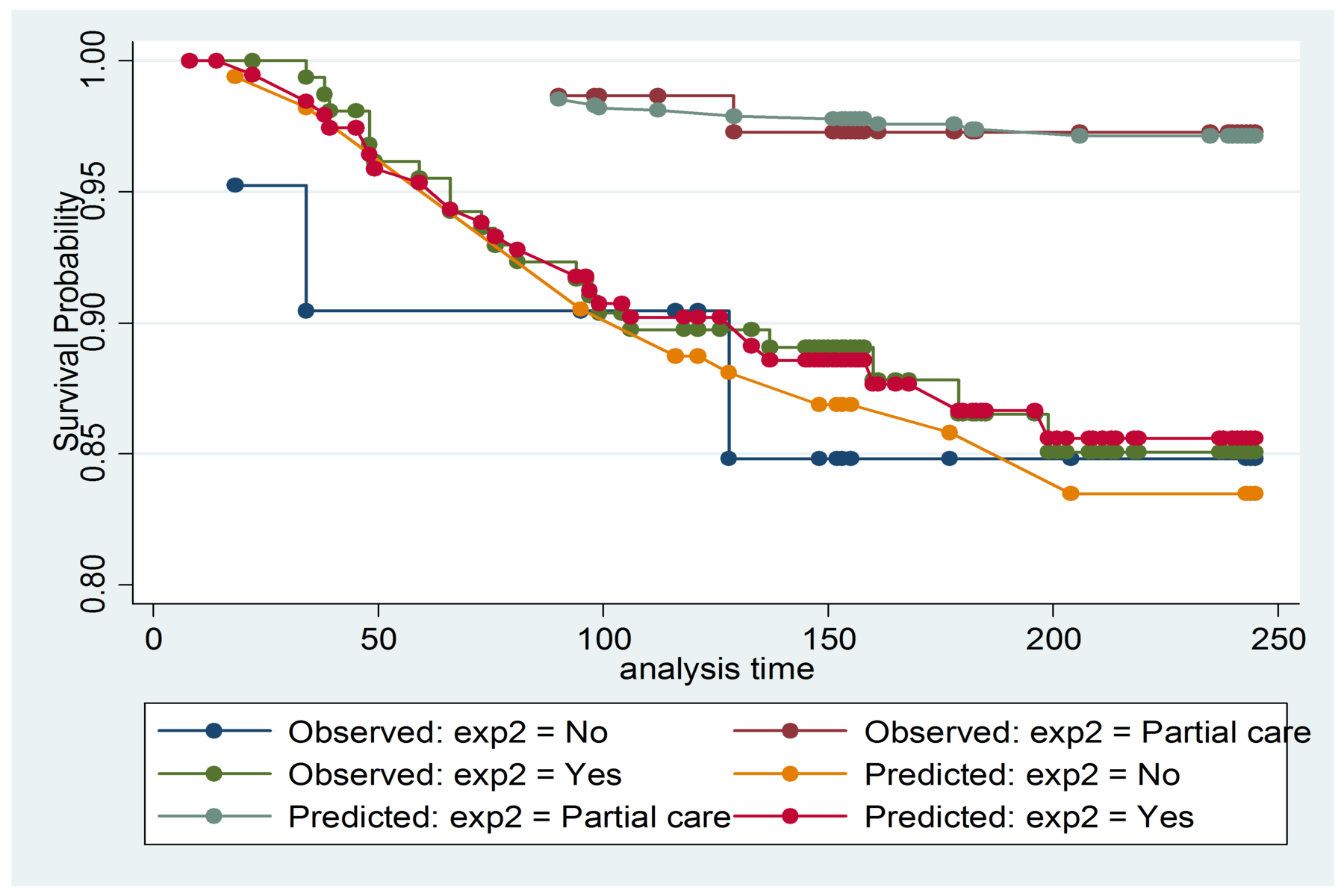

Supplement: S2 Fig — *exp2 variable categorized as (Yes) under care before treatment initiation; (Partial care) under care after treatment initiation; and (No) Not under care till declaration of outcomes; AIC / BIC: 265.7 / 272.8 (TIF) [file pone.0187223.s002.tif]
